# Activities and metabolomics of *Cordyceps gunnii* under different culture conditions

**DOI:** 10.3389/fmicb.2022.1076577

**Published:** 2023-01-12

**Authors:** Shuai-Ling Qu, Juan Xie, Jun-Tao Wang, Guo-Hong Li, Xue-Rong Pan, Pei-Ji Zhao

**Affiliations:** State Key Laboratory for Conservation and Utilization of Bio-Resources in Yunnan, School of Life Sciences, Yunnan University, Kunming, Yunnan, China

**Keywords:** *Cordyceps gunnii*, different growing conditions, untargeted metabolome, cytotoxic activity, nematocidal activity

## Abstract

Many active metabolites have been identified from various species of the fungal genus *Cordyceps*. A predominant species of this genus is *Cordyceps gunnii*, but there are limited reports on the active ingredients from this species. This study aimed to conduct activity assays and metabolome analysis on extracts of *C. gunnii* obtained under different culture conditions. Five different solid media were selected to culture the mycelium of *C. gunnii* and the metabolites were extracted with organic solvents; concurrently, the wild stroma and host complexes of *C. gunnii* were extracted by ethyl acetate. Extracts were subsequently assayed for various biological activities and were analyzed by untargeted metabolomics. There were significant differences in the activities and metabolites of *C. gunnii* extracts from different culture conditions and from wild stroma and host complexes. The extracts of stroma and host complexes and mycelia cultured on WGA medium for 21 days exhibited similar effective inhibitory activity against five cell lines. A total of 51 metabolites were annotated and included various structural types. The literatures indicate that most of the identified compounds have a variety of different biological activities. These findings provide the basis for further systematic excavation of *C. gunnii* and improved utilization of this fungal species.

## 1. Introduction

*Cordyceps* is a genus of fungi that are parasitic on insects, fungi, or plant bodies. According to the latest data from MycoBank,^1^ *Cordyceps* is the most abundant and diverse genus in the family Clavellaceae, and 630 species have been identified, with more than 130 of these species reported in China (Olatunji et al., 2018). Fungi of the genus *Cordyceps* (including stroma and host complexes, fruit body and mycelium) produce numerous secondary metabolites and have a variety of biological activities (Qu et al., 2022).

*Cordyceps gunnii* (heterotypic synonym: *Paecilomyces gunnii*), originally discovered in Tasmania, Australia, was first isolated and identified in China in Duyun county of Guizhou Province (Liang, 1983), and its activity and metabolites have attracted constant attention. Aqueous and alcoholic extracts of *C. gunnii* mycelium markedly reduced writhing times in mice using pain models of acetic acid-induced writhing and hot-plate tests in mice with intragastric administration and hypodermic injection (Chen et al., 2009). Se-polysaccharide obtained from selenium-enriched mycelia of *C. gunnii* exhibited anti-tumor activity within ovarian tumor model rats modeled with SK-OV-3 cells (Sun et al., 2018), while polysaccharides from *C. gunnii* mycelia showed immunomodulatory activity *via* the TLR4/NF-κB signaling pathway (Meng et al., 2019). Three novel macrocyclic tetrameric lactams—gunnilactams A, B, and C—were obtained from the submerged fermentation broth of *C. gunnii*, and gunnilactam A showed selective moderate cytotoxic activity against C42B cells (human prostate cancer) with an IC_50_ value of 5.4 μM (Zheng et al., 2017). However, some studies have reported that extracts from the mycelium of *C. gunnii* have no apparent activity. For example, Meng et al. (2012) found that water extracts and ethyl acetate extracts of *C. gunnii* cultured in potato-dextrose-broth (PDB) did not exhibit obvious activity against BEL-7402 cells and COLO205 cells.

In addition, different fermentation conditions had significant effects on the content and anti-tumor activity of polysaccharide from *C. gunnii* (Liu et al., 2019). In 2002, Zeeck coined the term OSMAC (One Strain Many Compounds) to describe the approach of modifying fermentation conditions to increase the types and abundance of microbial metabolites (Bode et al., 2002), and there has been some successful research using this strategy. For example, Paranagama et al. (2007) found that culture conditions affected the metabolite production of the fungi *Paraphaeosphaeria quadriseptata* and *Chaetomium chiversii*. The main reason for this may be that the metabolic biosynthesis genes are expressed differently under different growth conditions (Scherlach and Hertweck, 2009). As part of our ongoing search for undescribed active compounds from microorganisms, a series of novel active compounds were obtained from different fungi (Pu et al., 2021; Liu et al., 2022).

In the present work, five different solid media were selected to culture the mycelium of *C. gunnii* according to the literature and our preliminary experiments, and the metabolites were extracted with organic solvents; simultaneously, the metabolites of the wild stroma and host complexes of *C. gunnii* were extracted with ethyl acetate. The extracts were assayed for various activities and analyzed by untargeted metabolomics.

## 2. Materials and methods

### 2.1. Experimental strain and culture conditions

*Cordyceps gunnii* YMF1.00003 is preserved in the State Key Laboratory of Conservation and Utilization of Biological Resources in Yunnan and was identified as *Cordyceps gunnii* after internal transcribed spacer (ITS) identification (GenBank accession no. OP474063). Stroma and host complexes of *C. gunnii* were purchased from Shiqian County of Guizhou Province, and the ITS of the stroma was sequenced and identified (GenBank accession no. OP558778). *Meloidogyne javanica* was obtained from the roots of tomatoes grown in E’shan County in Yunnan Province. Eggs of *M. javanica* were acquired according to Liu et al. (2022). Briefly, *M. javanica* egg masses were handpicked from tomato root galls, surface-sterilized in 1% NaClO solution for 4 min, rinsed three times with distilled water (dH_2_O), placed in a Petri dish with water, and incubated in the dark at 25°C to prepare second-stage juveniles (J2s). The newly hatched J2s were collected daily. The nematode concentration was adjusted to 2 × 10^4^ nematodes/mL according to experimental needs.

Based on the literatures and our previous preliminary experiments, we selected nutrient-rich media, including various carbon sources, organic nitrogen sources, coenzymes and trace elements; and tried to make significant differences in the composition of these media. Finally, five media [CMA (20.0 g maize, 10.0 g glucose, 0.4 g peptone, 1.0 g VB, 1 l water); YMG (4.0 g yeast extract, 20.0 g glucose, 1 l water); PDA (200.0 g potato, 20.0 g glucose, 1 l water); GPY (20.0 g glucose, 6.0 g peptone, 10.0 g yeast paste, 1 l water); WGA (30.0 g wheat bran, 20.0 g glucose, 1.5 g KH_2_PO_4_, 1.5 g MgSO_4_, 1 l water)] were used to optimize culture conditions for mycelia of *C. gunnii*. Briefly, 200 ml each solid medium was divided into six Petri dishes. Then, the mycelium of *C. gunnii* is inoculated into each Petri dish and cultivated at 28°C for 14 or 21 days, respectively. The cultures were extracted exhaustively three times by EtOAc/MeOH/AcOH (80:15:5, by vol.). The soaking solution was obtained by filtration (repeated three times) and was evaporated under reduced pressure at 45°C to obtain the infusion. These extracts were named as CMA14, CMA21, YMG14, YMG21, PDA14, PDA21, GPY14, GPY21, WGA14, and WGA21, respectively. The stroma and host complexes of *C. gunnii* were cut and immersed in ethyl acetate organic solvent for 3 days, then filtrated to obtain the immersion solution (repeated three times); this extract was named CG. Finally, the extract was dried under reduced pressure at 45°C. All extracts were assayed for various biological activities and were analyzed by liquid chromatography-mass spectrometry (LC–MS).

### 2.2. 3-(4,5-dimethylthiazol-2-yl)-5-(3-carboxymethoxyphenyl)-2-(4-sulfophenyl)-2H-tetrazolium (MTS) method for cytotoxic activity

All extracts (100 μg/ml) were evaluated for their cytotoxicity activity by 3-(4,5-dimethylthiazol-2-yl)-2,5 diphenyl tetrazolium bromide (MTT) method. The MTT method was used for the bioassays was conducted as described in the literature (Su et al., 2013). Five cell lines were selected for testing (leukemia cell line HL-60, hepatocarcinoma cell line SMMC-7721, lung adenocarcinoma cell line A549, breast cancer cell line MDA-MB-231, and colon cancer cell line SW480). Taxol was used as a positive control. All experiments were performed in triplicate, and the data are expressed as means ± standard deviation (SD) of three independent experiments.

### 2.3. Assay for protective effect against corticosterone-induced oxidative stress

Poorly differentiated PC12 cells were maintained in DMEM medium supplemented with 10% fetal bovine serum (FBS), penicillin (100 U/ml), and streptomycin (100 μg/ml), and were incubated at 5% CO_2_ and 37°C. Subsequently, the cells were divided into the following groups: Blank group (contains PC12 cells and DMSO at a final concentration of 0.1%); NC group (contains PC12 cells, a final concentration of 150 μM corticosterone (CORT), and a final concentration of 0.1% DMSO); DIM group [contains PC12 cells, a final concentration of 10 μM desipramine (DIM), a final concentration of 150 μM CORT, and a final concentration of 0.1% DMSO]; Each extracts group (contains PC12 cells, a final concentration of 150 μM CORT, and 20 μg/ml extract). Briefly, poorly differentiated PC12 cells were seeded in 96-well culture plates at a density of 1 × 10^4^ cells/well. After 24 h, samples were added to the wells according to the previously described groups and were incubated for 48 h. MTS solution was then added to each well and the absorbance was measured at 492 nm using a Thermo Multiskan FC. Each group included three repetitions (Jiang et al., 2014).

### 2.4. Assay for nematocidal activity against *Meloidogyne javanica*

The tested extracts were dispersed in MeOH. Two hundred *M. javanica* J2s (100 μl) were added to each sample, and the final concentration of the tested compounds was set at 10 mg/ml. The total and dead nematode numbers were enumerated every 24 h (Huang et al., 2020); nematodes were considered dead if they were flat or cracked. Subsequently, nematode mortality was calculated. Avermectin was used as a positive control, and test solution without compound was used as a negative control. Three replicates were conducted for each test.

### 2.5. Metabolomic data acquisition and statistical analysis

Untargeted LC–MS metabolomics was performed on a Dionex UltiMate 3,000 LC system coupled with a Q-Exactive Orbitrap MS (Thermo, San Jose, CA, United States). All samples were separated on an Agilent Zorbax Eclipse Plus C18 (50 × 2.1 μm; Agilent Technologies, CA, United States) with a particle size of 1.8 μm at an LC flow rate of 300 μl/min and a column temperature of 40°C. Mobile phase A comprised water containing 0.5% formic acid, and mobile phase B comprised 0.5% formic acid in methanol. The extracts were prepared separately by dissolving in chromatographic methanol to a concentration of 10 mg/ml, filtering three times, placing at 4°C overnight, and then setting aside as samples for LC–MS detection. The 30-min gradient for positive electrospray ionization (ESI) mode was set as: 0–3 min, 1% solvent B; 3–20 min, 1–99% solvent B; 22–25 min, 99% solvent B; and 25–30 min, 1% solvent B. The injection volume was 5 μl, and each sample was injected in triplicate. The injection order was randomized, and the group information was blinded for LC–MS analysis. The instrument settings included a capillary temperature of 350°C, sheath gas flow rate of 35 (arbitrary units), auxiliary gas flow rate of 10 (arb), spray voltage of 4.0 kV, full MS resolution of 70,000, and MS/MS resolution of 17,500. Each sample was prepared in biological triplicate. The LC–MS instrument was controlled using Thermo Scientific Xcalibur 4.1 software.

The raw data file was analyzed using Compound Discoverer (CD version 3.3, Thermo Fisher Scientific) software for metabolomics data analysis. A blank sample was used for background subtraction and noise removal during the pre-processing step. The data were analyzed in 11 groups (CG, CMA-14, CMA-21, YMG-14, YMG-21, PDA-14, PDA-21, GPY14, GPY21, WGA-14, and WGA-21). For analysis of the data on metabolite variation in the nine groups, simple univariate statistical analyses were carried out on log_2_-transformed data using a paired *t*-test. Volcano plots were created using these data, with a threshold of *p* < 0.05 and absolute log_2_ fold-change of >1 set for defining a notable change in compound abundance among all samples. All components among were searched against an accurate mass database consisting of known fungal metabolites using a mass tolerance of 10 ppm. The database was prepared using SciFinder, and additional fungal natural products were identified in the literatures. Meanwhile, other potential compound identifications were obtained by comparing the MS/MS scan with the MZCloud, ChemSpider, and MZvault libraries. To confirm and evaluate intact mass-based identifications, manual analyses of fragmentation data were performed as described below. All compounds tentatively identified *via* accurate intact mass were confirmed using accurate mass, tandem MS (MS^2^) data. To ensure that low-quality spectra were not included, MS^2^ spectra containing fewer than five peaks at >1% relative abundance were excluded from the analysis. Additionally, spectra containing more than 100 peaks at >1% abundance were included only if >20% of the peaks appeared in the higher m/z half of the spectrum. Both general fragmentation rules and fragmentation library modes were used. When published fragmentation data were available, a comparison was also performed to further confirm identifications.

## 3. Results and discussion

### 3.1. Stroma and host complex and culture status of *Cordyceps gunnii*

**Figure 1:**
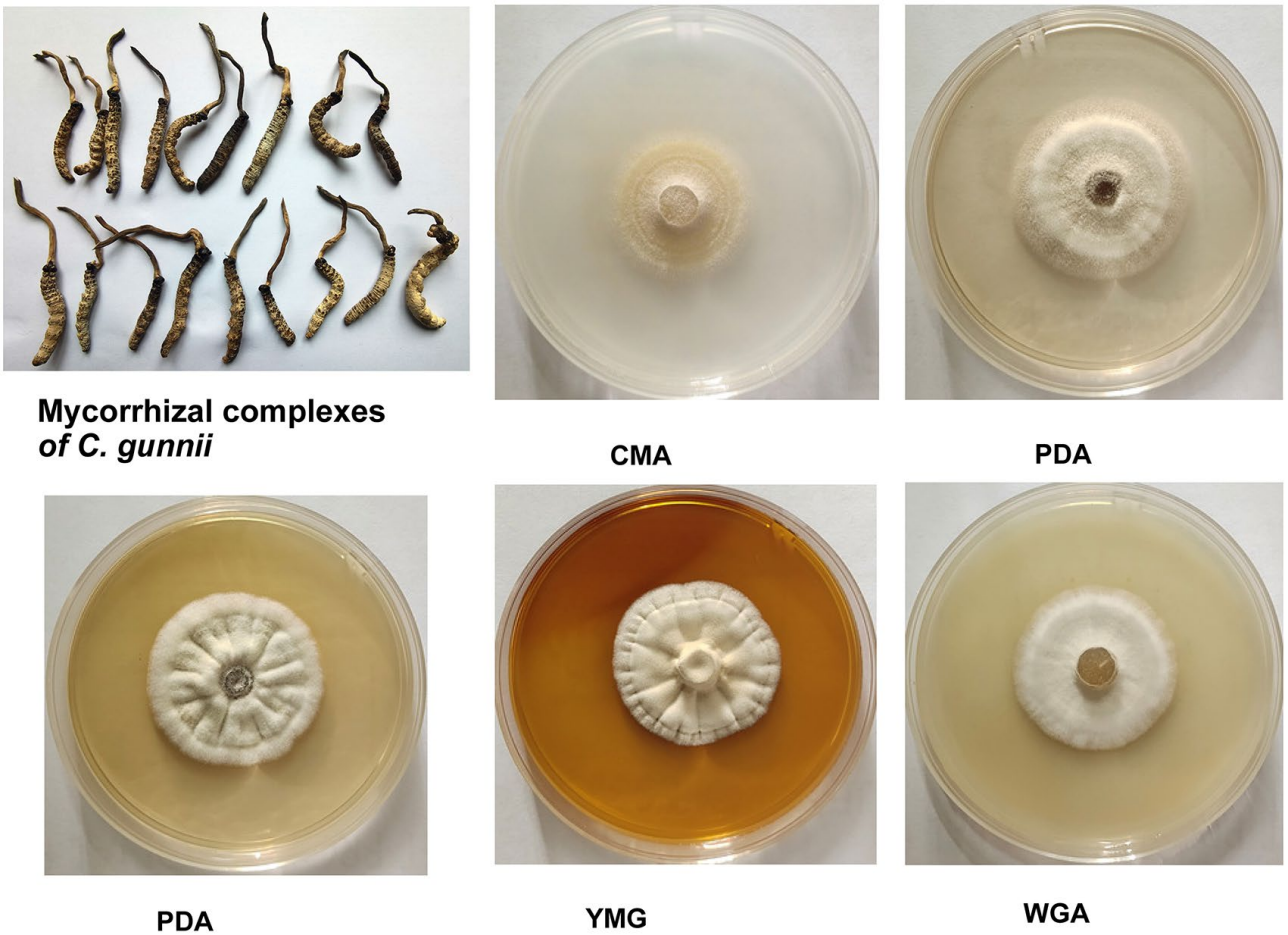
Appearance of solid-state fermentation cultures of *Cordyceps gunnii* after 21 days on different media, and stroma and host complexes of *C. gunnii*.

In the stroma and host complex, the host was white, gray to brown (dried specimen), without hyphae on the surface; the stroma was single, yellow to brown, and rose from the head of the host (Figure 1). Colony diameters were 21–23 mm and 22–27 mm on CMA after incubation at 28°C for 14 and 21 days, respectively. The colonies were dense, white at first, then turning pale to light yellow. Colonies on PDA were circular, white, reverse yellow and attained diameters of 20–26 and 29–33 mm at 28°C after 14 and 21 days, respectively. Synnemata emerged from the surface and in the margin of the colony and were pale yellow to yellow. Colonies grown on YMG and GPY were radiological with diameters the same as those on PDA, and were dense and white to pale yellow. Colonies grown on WGA were circular, white, and attained diameters of 20–22 and 26–30 mm after growth at 28°C for 14 and 21 days, respectively.

To quantify the various extracts in different medium, culture and extraction was performed. Hundred milliliter of each of the five media (GPY, CMA, YMG, WGA, and PDA) divided into 5 Petri dishes plates, respectively; then the mycelium was inoculated on the media and incubated for 14 or 21 days, respectively. After 21 days of mycelium growth, the weights of the five extracts GPY-21, CMA-21, YMG-21, WGA-21, and PDA-21 were 346, 286, 191, 390, and 193 mg, respectively.

### 3.2. Cytotoxic activity of extracts

**Figure 2:**
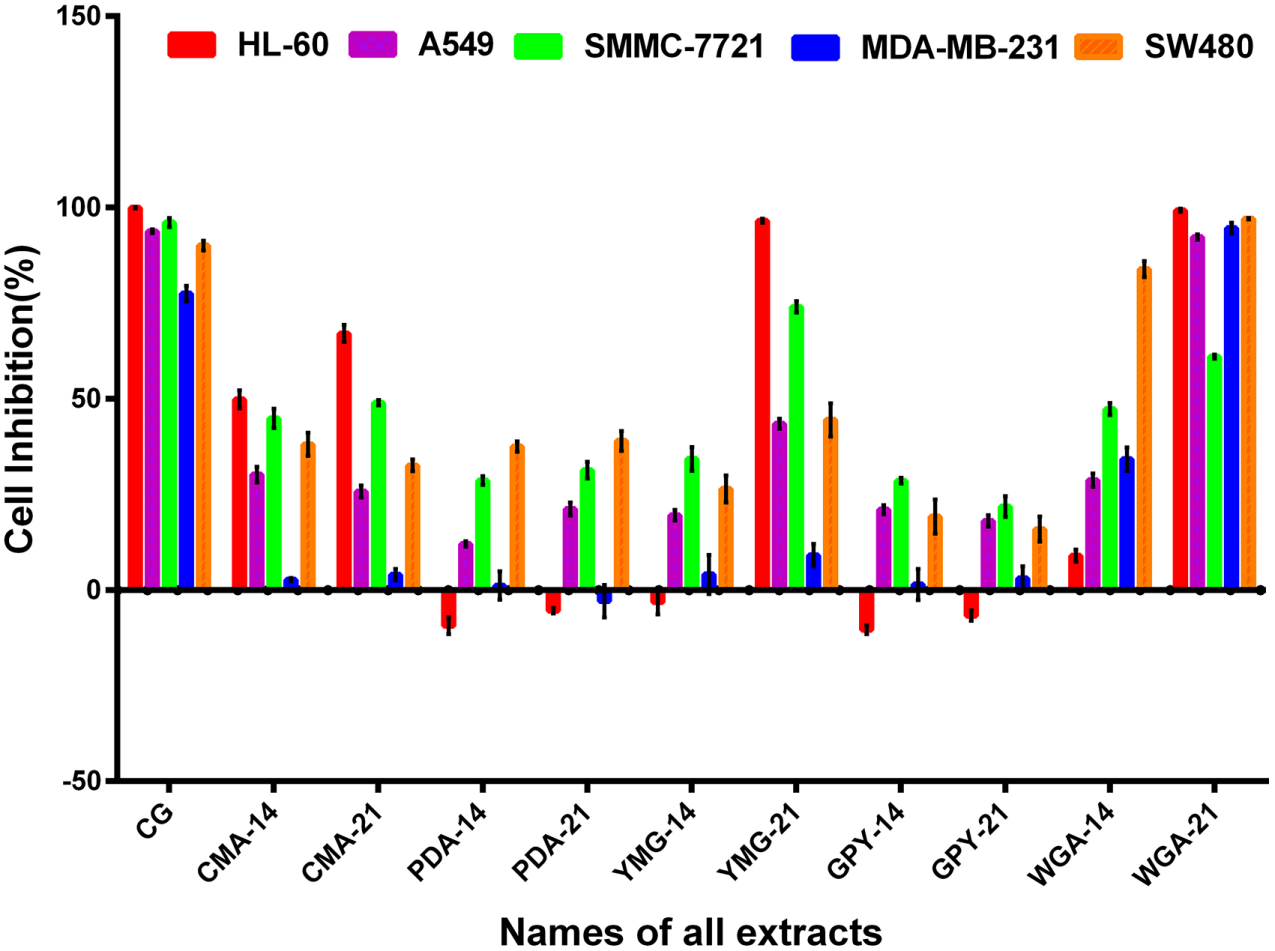
The cytotoxic activity of the crude extract.

The cytotoxic activity of the extracts of *C. gunnii* cultured under different conditions was examined against five human cancer cell lines (HL-60, A549, SMMC-7721, MDA-MB-231, and SW480). All extracts were tested at a concentration of 100 μg/ml, and Taxol was used as a positive control. As shown in Figure 2, the cytotoxic activity of the extracts from different culture conditions and stroma and host complexes exhibited significant differences. The CG group, extracted from stroma and host complexes of *C. gunnii*, showed strong inhibitory activity against the five tumor cell lines. The extracts from the mycelium of *C. gunnii* cultured for only 14 days had no significant cytotoxic activity at the tested concentrations except that WGA-14 group showed selective inhibition of the SW480 cell line. However, the cytotoxic activity of the extracts from mycelium cultured for 21 days was very different. Among them, WGA-21 group had marked inhibitory activity against all five cell lines, which was almost similar to the CG group. YMG-21 group was the most effective extract in HL-60 and SMMC-7721 cells, while the CMA-21 group exhibited certain inhibitory activity on HL-60 cells.

### 3.3. Protective activity of extracts against neural cell damage induced by corticosterone

CORT-induced PC12 cell damage is used as an *in vitro* experimental model for depression studies. All extracts were evaluated for their protective activities against PC12 cell injury induced by CORT. After adding the extracts for 48 h, the absorbance of each well was measured by the MTS method and the cell survival rate was calculated. None of the 11 extracts had any apparent protective effect on corticosterone-induced nerve cell damage at the concentration of 20 μg/ml (Supplementary Figure S1).

### 3.4. Nematocidal activity of extracts against *Meloidogyne javanica*

**Figure 3:**
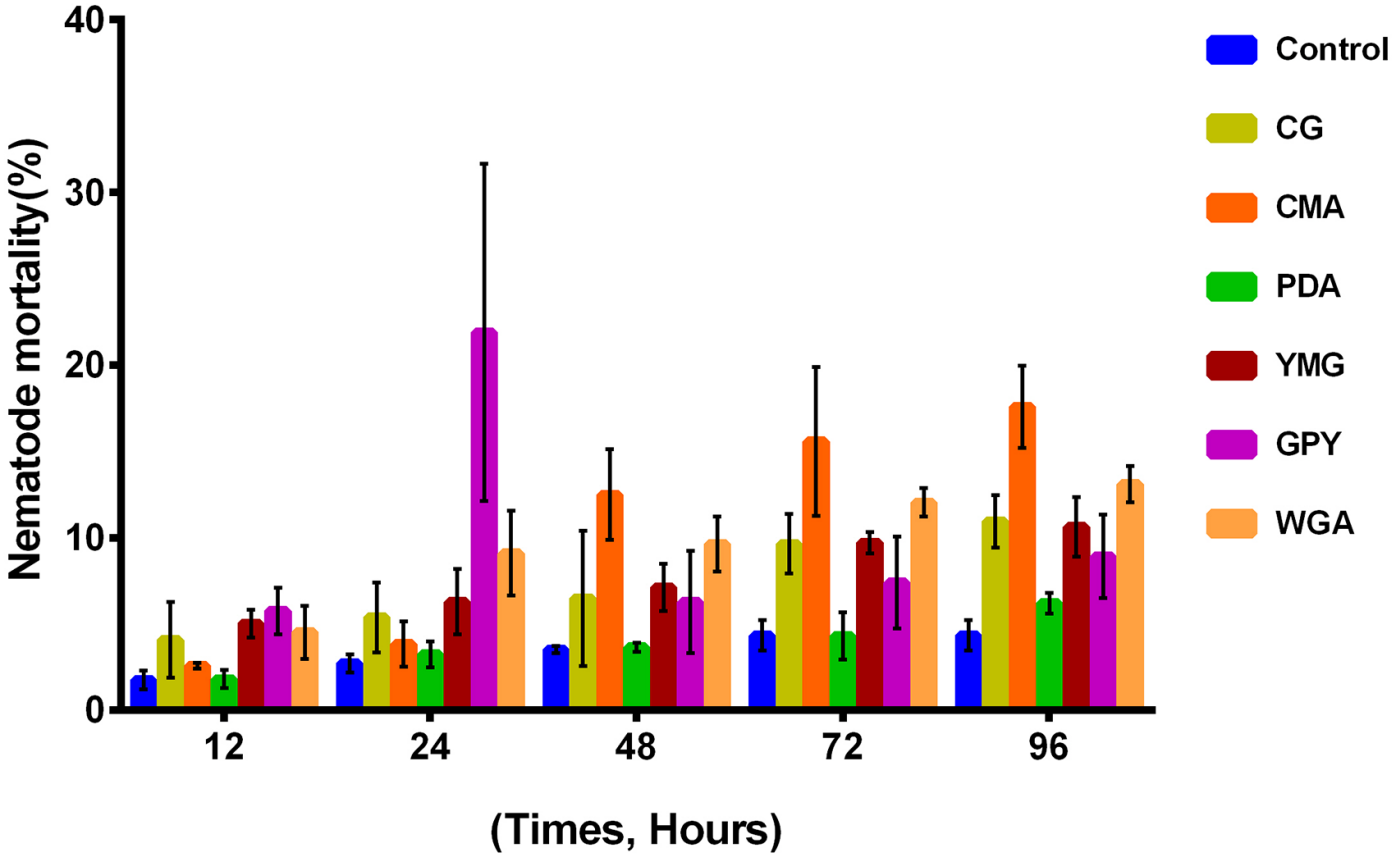
Nematocidal activity of the crude extracts of *C. gunnii*.

The nematocidal activity of the crude extracts of *C. gunnii* was assayed against *M. javanica* by counting the number of dead nematodes at 12, 24, 48, 72, and 96 h in the presence of 10 mg/ml crude extracts. None of the extracts showed significant activity. The highest nematocidal activity was observed with the extract from CMA medium (Figure 3), but the mortality was only 18.38% at 96 h. In addition, the GPY extract showed a paralyzing effect on *M. javanica* at 24 h, with 20–30% nematodes in a state of paralysis; however, at subsequent time points those nematodes were restored.

### 3.5. Untargeted metabolomics analysis of extracts

**Figure 4:**
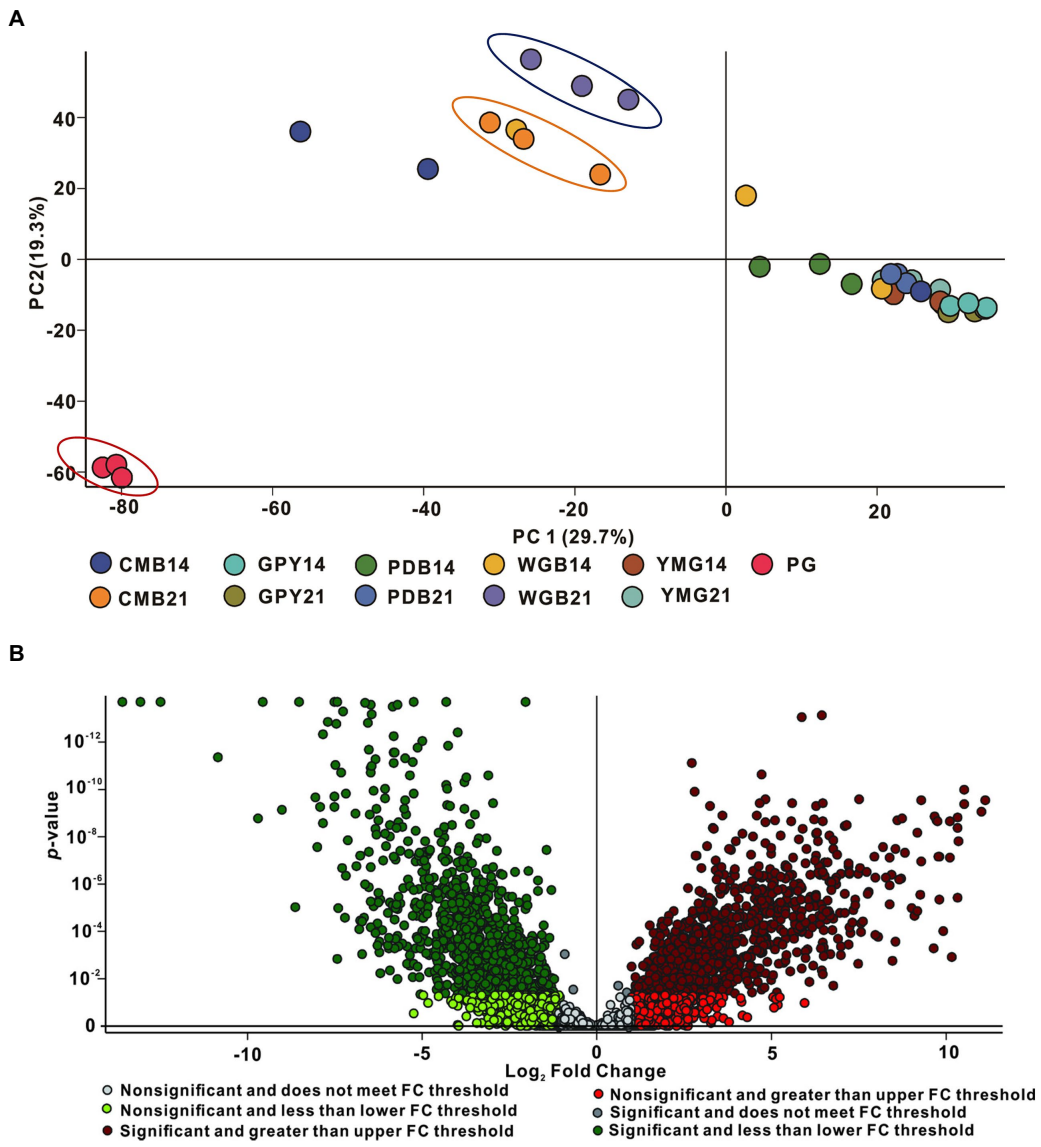
Principal component analysis (PCA) and volcano plot of all samples. **(A)** PCA of all extracts. **(B)** The volcano plot of CG group vs. WGA group.

To determine whether metabolites and metabolic pathways in *C. gunnii* were changed under different culture conditions, the extract samples were subjected to LC–MS untargeted metabolomics. Quantitative analysis of low-molecular-weight metabolites can reveal the relative relationship between changes and metabolites, and may indicate the reasons for differences in activity in the extracts from different culture media. Extracted metabolites were analyzed in positive ion modes as described in the section “Materials and Methods.” Loading data for Principal Component Analysis (PCA) was derived from all metabolites identified by Compound Discoverer 3.3 after LC–MS analysis and their peak area tendencies. The first and second principal components (PC1 and PC2) explained 49% of the overall variance. CG was significantly separated from all other samples in PC1 (29.7%) and PC2 (19.3%) (Figure 4A). CMA-21 was clearly separated from WGA-21 and other samples. Furthermore, the separation degree of WGA-21 and CMA-21 was relatively close to CG, while other samples (CMA-14, YMG-14, YMG-21, PDA-14, PDA-21, and WGA-14) showed no obvious separation. This result highlights the significant differences in the major metabolites produced by *C. gunnii* under different culture conditions. Referring to the results of the activity assays, subsequent work focused on identifying differences in the compounds produced in WGA-21 and CG.

Combining all the analyzed extracts, 4,382 unique molecular species were detected using UPLC-HR-ESI-MS. The high-resolution MS signals from different isotopes and adduct peaks were combined so that the vast majority of molecular species represented individual metabolites produced by the corresponding strain. We aimed to determine the differences in secondary metabolites between CG and WGA groups, and data were displayed as a volcano plot for visualization. Using significance cutoffs of a false discovery rate (FDR)-adjusted *p-*value (<0.05) and a fold-change difference > 1, 886 metabolites were upregulated in the CG group, while 879 metabolites were upregulated in the WGA group (Figure 4B). To determine the structures of the upregulated compounds, a structural library containing 249 metabolites from the genus *Cordyceps* was assembled after removing common steroids and fatty acids (Olatunji et al., 2018; Qu et al., 2022).

The complete masses of upregulated compounds were used to search the structural library and other libraries (MZCloud, ChemSpider, and MZvault). When searching these databases with a mass tolerance of 10 ppm, 51 annotations were verified (Table 1). The 51 identified compounds include various structural types such as non-ribosomal peptide synthetase (NRPS), polyketides (PKS), terpenes, and nucleosides. Among the 51 compounds, 19 were unique to the WGA group, 18 were unique to the CG group, and the other 14 compounds were present in the extracts of both WGA and CG groups. Most of these compounds were derived from the genus *Cordyceps* and other fungi, and a wide variety of activities have been reported. Furthermore, there were numerous metabolites with undefined structures that were significantly upregulated in the CG and WGA groups. The screening of culture conditions based on activity and metabolome is a powerful tool to facilitate exploration of the active metabolites of *Cordyceps*.

**Table 1:** Known compounds confidently identified from extracts of the WGA and CG groups.

Identified metabolites	m/z	Formula	Ion mode	Cal. mass	Delta mass (ppm)	Distribution
GameXPeptide F	615.4199	C_33_H_54_N_6_O_5_	[M + H]^+^	615.4229	−4.79	Both
Pestalotiopin B	544.3611	C_32_H_49_NO_6_	[M + H]^+^	544.3633	−4.46	Both
Fumosoroseain A	498.3766	C_28_H_52_NO_6_	[M + H]^+^	498.3789	−2.24	WGA
Me lucidenate N	475.3035	C_28_H_43_O_6_	[M + H]^+^	475.3054	−1.75	CG
Certonardosterol J	463.3767	C_29_H_51_O_4_	[M + H]^+^	463.3782	−1.45	CG
3,5-Dihydroxy-14,15-epoxyergosta-7,22-diene-6-one	443.3136	C_28_H_42_O_4_	[M + H]^+^	443.3156	−2.02	CG
27-*O*-methylasporyzin C	436.3194	C_29_H_42_NO_2_	[M + H]^+^	436.321	−1.96	WGA
4′-Hydroxylisoflavone-7-*O*-*β*-4′′-methoxylglucopyranoside	431.1316	C_18_H_18_N_6_O_7_	[M + H]^+^	430.131	0.66	WGA
7-Ketositosterol	429.3709	C_29_H_49_O_2_	[M + H]^+^	429.3727	−1.84	CG
Ergosterol peroxid	429.3343	C_28_H_45_O_3_	[M + H]^+^	429.3363	−2.04	WGA
3-Hydroxy-5,9-epoxy-7,22-dien-6-one-ergosta	427.3194	C_28_H_43_O_3_	[M + H]^+^	427.3207	−1.26	CG
3-Oxoergosta-1,4,22-trien-26-oic acid	425.3031	C_28_H_41_O_3_	[M + H]^+^	425.3031	2.31	Both
*N6*-(Glycyl-l-glutaminyl)-d-lysyl-d-alanine	425.2126	C_16_H_30_N_6_O_6_Na	[M + Na]^+^	425.2119	0.66	CG
Dihydrobrassicasterol	423.3605	C_30_H_47_O	[M + H]^+^	423.3621	−1.59	Both
Glycoasperfuran	417.1525	C_18_H_21_N_6_O_6_	[M + H]^+^	417.1517	0.79	WGA
*N*-oleoyl-l-glutamine	411.3237	C_23_H_43_N_2_O_4_	[M + H]^+^	411.3258	1.93	CG
Campesterol	401.3718	C_28_H_49_O	[M + H]^+^	401.3778	−6	WGA
Ergosta-5,7,22,24 (28)-tetraen-3-ol	395.3290	C_28_H_43_O	[M + H]^+^	394.3308	−1.8	CG
Ergosta-4,6,8 (14),22E-tetraen-3-one	393.3137	C_28_H_41_O	[M + H]^+^	393.3152	−1.48	CG
12-(3-adamantan-1-ylureido)dodecanoic acid	393.3135	C_23_H_41_N_2_O_3_	[M + H]^+^	393.3112	2.31	CG
Cordypyrone B	379.1898	C_22_H_28_O_4_Na	[M + Na]^+^	379.188	1.77	WGA
Opaliferin	375.1429	C_18_H_24_O_7_Na	[M + Na]^+^	375.1414	1.48	WGA
3,4-Diacetoxy-12,13-epoxy-9-trichothecene-15-ol	367.1714	C_19_H_27_O_7_	[M + H]^+^	366.1751	−3.68	Both
Cordypyrone A	363.1945	C_22_H_28_O_3_Na	[M + H]^+^	363.1931	1.47	CG
4-Acetoxyscirpene-3,15-diol	325.1611	C_17_H_25_O_4_	[M + H]^+^	325.1646	−3.42	Both
Palythinol	325.1244	C_14_H_22_O_7_Na	[M + H]^+^	325.1258	−1.41	WGA
Ovalicin	319.1507	C_16_H_24_O_5_Na	[M + H]^+^	319.1516	−0.15	Both
Annullatin C	317.1712	C_17_H_26_O_4_Na	[M + H]^+^	317.1723	−1.09	CG
Cordycepone	311.1249	C_17_H_20_O_4_Na	[M + H]^+^	311.1254	−0.44	CG
2′-Deoxy-5′-uridylic acid	311.0635	C_9_H_16_N_2_O_8_P	[M + H]^+^	311.0639	−0.36	CG
Lupinic acid	307.1505	C_13_H_19_N_6_O_3_	[M + H]^+^	307.1513	0.98	Both
Mesterolone	305.2462	C_20_H_33_O_2_	[M + H]^+^	305.2473	−1.31	CG
Paecilomycine B	305.1347	C_15_H_22_O_5_Na	[M + Na]^+^	305.1359	−1.27	Both
Annullatin B	301.1783	C_17_H_26_O_3_Na	[M + Na]^+^	300.1774	0.87	Both
Annullatin A	299.1605	C_17_H_24_O_3_Na	[M + Na]^+^	299.1594	−1.31	Both
2-Amino-2′-*O*-methyladenosine	297.1318	C_11_H_17_N_6_O_4_	[M + H]^+^	297.1306	1.22	CG
2′-Amino-2′-deoxyguanosine	283.1162	C_10_ H_14_N_6_O_4_	[M + H]^+^	283.1149	1.32	WGA
Paecilomycine A	267.1578	C_15_H_22_O_4_	[M + H]^+^	267.1591	−1.32	WGA
Amdoxovir	253.1035	C_9_H_12_N_6_O_3_	[M + H]^+^	253.1044	−0.83	WGA
Cordycepin	252.107	C_10_H_14_N_5_O_3_	[M + H]^+^	252.1091	−2.17	WGA
Cyclo(l-Phe-l-Pro)	245.1273	C_14_H_17_N_2_O_2_	[M + H]^+^	245.1285	−1.18	Both
Lumichrome	243.0866	C_12_H_10_N_4_O_2_	[M + H]^+^	243.0877	−1	WGA
8-(Hydroxyethylamino)adenine	227.1244	C_8_H_13_N_6_O_2_	[M + H + MeOH]^+^	227.1251	−0.76	CG
2,6-Diamino-9-(2-hydroxyethoxymethyl)purine	225.1087	C_8_H_13_N_6_O_2_	[M + H]^+^	225.1095	−0.7	CG
Cephalosporolide C	217.1061	C_10_H_17_O_5_	[M + H]^+^	217.1071	−0.93	WGA
Cyclo(Leu-Pro)	211.1430	C_11_H_19_N_2_O_2_	[M + H]^+^	211.1414	−1.1	Both
2,6-Dihydroxypseudooxynicotine	211.1068	C_10_H_15_N_2_O_3_	[M + H]^+^	211.1077	−0.94	WGA
Cepharosporolide E	199.0956	C_10_H_15_O_4_	[M + H]^+^	199.0965	−0.93	WGA
4,7-Dihydroxyoct-2-enoic acid	197.0799	C_8_H_14_O_4_Na	[M + Na]^+^	197.0784	1.5	WGA
Cyclo(valyl-prolyl)	197.1275	C_10_H_17_N_2_O_2_	[M + H]^+^	197.1285	−0.75	Both
Suspensolide	195.1371	C_12_H_19_O_2_	[M + H]^+^	195.138	−0.86	WGA

Of the 51 annotated compounds, many are reported to show a wide variety of biological activities. For example, two sesquiterpenoids, 3,4-diacetoxy-12,13-epoxy-9-trichothecene-15-ol and acetoxyscirpenediol, showed significant cytotoxic activity (Claridge et al., 1979; Nam et al., 2001); and simultaneously, this type of compound also has good nematicidal activity. Ovalicin and a related compound showed anti-angiogenic activity (Pillalamarri et al., 2019) and cytotoxicity (Chang et al., 2013). Opaliferin exhibited slight cytotoxicity (Grudniewska et al., 2014). In addition, cordycepin is the most famous active metabolite from *Cordyceps*, and has a wide spectrum of activities (Qu et al., 2022). Amdoxovir is a nucleoside compound with significant antiviral properties (Quan and Peters, 2004). Annotation of these compounds in the metabolome can appropriately help explain the activity of the extracts. In addition, because the compound(s) in the crude extracts have some cytotoxic activity, some experiments can only be performed with a lower concentration of the extract, resulting in the inability to obtain improved experimental results. For example, the protective activity of corticosterone against neural cell damage requires the testing sample to not have obvious cytotoxic activity.

With the rapid development of sequencing technology, increasing numbers of complete microbial genomes have been reported. Analysis of most of the reported fungal genome data revealed that a large number of secondary metabolic gene clusters were silent (or weakly expressed) under experimental culture conditions, and the corresponding metabolites could not be isolated and identified (Brakhage and Schroeckh, 2011; Keller, 2019; Lei and Zhao, 2019). The culture conditions of microorganisms are known to be critical for the quantity and abundance of secondary metabolites in microbial fermentation studies. In recent years, through the OSMAC strategy, a series of compounds with novel structures and multiple activities have been isolated and identified from fungi. After screening the medium of *Stereum hirsutum*, four sesquiterpene and amino acid hybrid quaternary ammonium salts, stereumamides A–D, were identified from the fermentation products. These compounds have certain antibacterial activity (Duan et al., 2018). Meng et al. (2016) obtained the diketopiperazine compounds spirobrocazine A and brocazine G from the marine endophyte *Penicillium brocae* MA-231, and brocazine G has strong anti-*Staphylococcus aureus* activity [minimum inhibitory concentration (MIC) 0.25 μg/ml] and cytotoxic activity (IC_50_ of 664 and 661 nM against cell lines A2780 and A2780 CisR, respectively). Wakefield and colleagues co-cultured marine-derived *Aspergillus fumigatus* MR2012 with *Streptomyces leeuwenhoekii* strain C34 and strain C58, respectively, and found that the metabolites changed significantly. Among the co-cultured metabolites of *Aspergillus fumigatus* MR2012 and *Streptomyces leeuwenhoekii* C34, two novel compounds—luteoride D and pseurotin G—were isolated and identified. In addition, a lasso peptide (chaxapeptin), which was not detected in *Streptomyces leeuwenhoekii* C34, was also isolated under co-cultivation conditions (Wakefield et al., 2017). Another study used a similar approach to rapidly search for anti-COVID-19 natural products. The soybean-associated endophytic fungi *Aspergillus terreus* was cultured and screened in five media, and a total of 18 compounds were identified through metabolome analysis. Multivariate analysis subsequently showed that *Aspergillus terreus* was more suitable for producing metabolites growing in PDB and modified PDB, and molecular docking studies revealed that the metabolites aspergillide B1 and 3α-hydroxy-3,5-dihydromonacolin L may have high inhibitory activity against COVID-19 (El-Hawary et al., 2021).

## 4. Conclusion

Genomic data of *C. gunnii* has not yet been reported or released; therefore it is currently impossible to analyze potential secondary metabolites of this fungal species through genomic analysis. Early folk use of the stroma and host complexes of *C. gunnii*, so we look forward to find the active ingredients of *C. gunnii* by comparing the activities and metabolites of the mycelium under different cultural conditions with the active and metabolic components of *C. gunnii* in the wild. The activity and metabolome data of each extract varied significantly, among which the activities (cytotoxic and nematicidal activities) and metabolome data from WGA extract and wild *C. gunnii* were more similar. In addition to the compounds we annotated, a large number of unknown metabolites were produced under different growth and cultural conditions of *C. gunnii,* suggesting that the metabolites of this entomogenous fungus have the potential to be further explored.

## Data availability statement

The original contributions presented in the study are included in the article/Supplementary material, further inquiries can be directed to the corresponding author.

## Author contributions

P-JZ and G-HL: conceptualization and writing – review and editing. P-JZ, S-LQ, and JX: methodology. P-JZ and X-RP: software. P-JZ: validation and funding acquisition. P-JZ and S-LQ: data curation. S-LQ, JX, and J-TW: writing—original draft preparation. All authors have read and agreed to the published version of the manuscript.

## Funding

This research was funded by the National Natural Science Foundation of China (32270132 and 31970060) and Yunnan Science and Technology Special Project (202102AA100013 and 202001BB050061).

## Conflict of interest

The authors declare that the research was conducted in the absence of any commercial or financial relationships that could be construed as a potential conflict of interest.

## Publisher’s note

All claims expressed in this article are solely those of the authors and do not necessarily represent those of their affiliated organizations, or those of the publisher, the editors and the reviewers. Any product that may be evaluated in this article, or claim that may be made by its manufacturer, is not guaranteed or endorsed by the publisher.

## Supplementary material

The Supplementary material for this article can be found online at: https://www.frontiersin.org/articles/10.3389/fmicb.2022.1076577/full#supplementary-material

## Footnotes

^1^ https://www.Mycobank.org
