# Supplementary material for: Activities and metabolomics of Cordyceps gunnii under different culture conditions
Source: Front Microbiol. 2023 Jan 12;13:1076577. doi: 10.3389/fmicb.2022.1076577 (PMC9878563; doi:10.3389/fmicb.2022.1076577)
Supplement: Supplementary file 1 [file Data_Sheet_1.docx]

Supplementary Material

Activities and metabolomics of *Cordyceps gunnii* under different culture conditions

Shuai-Ling Qu^†^, Juan Xie^†^, Jun-Tao Wang, Guo-Hong Li, Xue-Rong Pan and Pei-Ji Zhao*

^1^State key Laboratory for Conservation and Utilization of Bio-Resources in Yunnan, School of Life Sciences, Yunnan University, Kunming, Yunnan 650091, China

**†** These authors contributed equally to this work and share first authorship

*** Correspondence:** Pei-Ji Zhao, Email: [pjzhao@ynu.edu.cn](mailto:pjzhao@ynu.edu.cn); Tel: 086-871-65031092

# Supplementary Figures

| 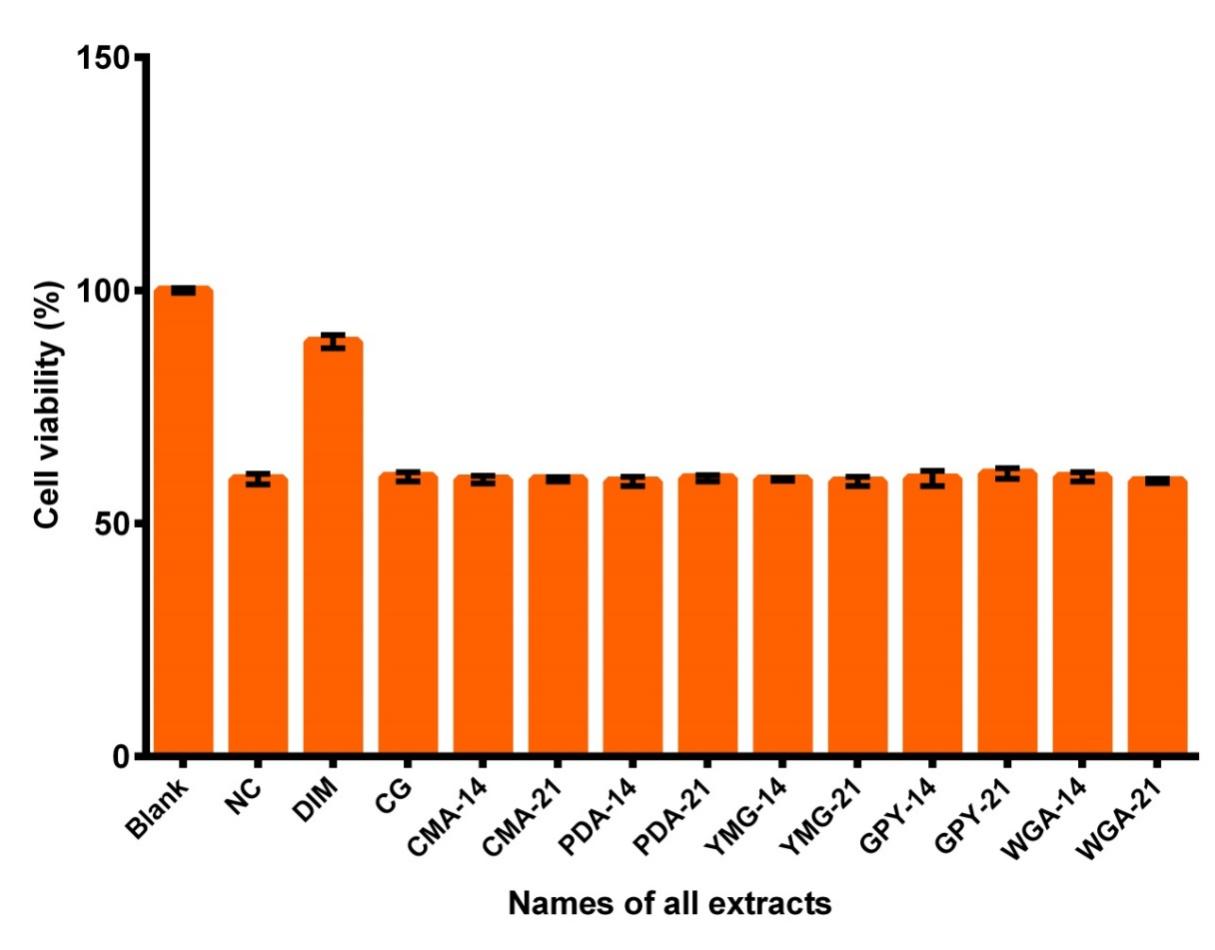  **FIGURE S1** Neuroprotective activity of CORT-induced PC12 cell damage by the crude extracts of *C. gunnii*. |
| --- |
